# EGFR Phosphorylates and Associates with EFNB1 to Regulate Cell Adhesion to Fibronectin

**DOI:** 10.1016/j.mcpro.2025.101027

**Published:** 2025-07-04

**Authors:** Ana I. Osornio-Hernández, François J.M. Chartier, Tim L. Schuehle, Sara L. Banerjee, Sabine Elowe, Patrick Laprise, Andrew Freywald, Mélanie Laurin, Nicolas Bisson

**Affiliations:** 1 Department of Molecular Biology, Medical Biochemistry and Pathology, Faculté de Médecine, Université Laval, Québec, Quebec, Canada; 2 Centre de recherche du Centre Hospitalier Universitaire (CHU) de Québec-Université Laval, Division Oncologie, Québec, Quebec, Canada; 3 PROTEO and Centre de recherche sur le cancer de l’Université Laval, Québec, Quebec, Canada; 4 Department of Pediatrics, Université Laval, Québec, Quebec, Canada; 5 Department of Pathology and Laboratory Medicine, College of Medicine, University of Saskatchewan, Royal University Hospital, Saskatoon, Saskatchewan, Canada

## Abstract

Ephrin-Bs (EFNB1-3) are ligands for members of the largest subfamily of receptor tyrosine kinases (RTKs) in humans, the EPH receptors. Interestingly, ephrin-Bs are transmembrane proteins that may also act as receptors themselves upon EPH binding, activating so-called reverse signaling pathways that are critical for multiple cellular processes. Although a number of ephrin-B signaling effectors have been identified, the molecular mechanisms underlying ephrin-B-driven cellular processes remain unresolved, suggesting that multiple signaling effectors are yet to be discovered. Here, we employed proximity labeling proteomics to delineate the proximity network of EFNB1 in steady state and under active reverse signaling conditions. This allowed us to identify 90 uncharacterized EFNB1 proximity partners, from which we could distinguish three main groups: EPH receptor stimulation-dependent, stimulation-independent, and negatively modulated by EPH receptor stimulation. We further investigated the functional relationship between EFNB1 and one of the candidates identified, the epidermal growth factor receptor (EGFR). We found that EFNB1 and EGFR associate in cells and showed that the formation of this complex relies on EFNB1’s PDZ-binding motif (PBM). Strikingly, we demonstrate that EGFR directly phosphorylates tyrosine residues within EFNB1’s PBM, which results in the disruption of the EFNB1-EGFR complex. Furthermore, we show that the EFNB1-EGFR association is required for EFNB1-dependent cell adhesion to fibronectin. Taken together, our results shed light on a functional relationship between EFNB1 and EGFR.

## Highlights

• Proximity labeling of EFNB1 reveals 90 previously uncharacterized proximity partners.
• EGFR is required for EFNB1-dependent cell adhesion to fibronectin.
• EGFR associates with EFNB1 *via* EFNB1’s PDZ-binding motif (PBM).
• EGFR phosphorylates EFNB1’s PBM on two tyrosine residues.
• Phosphorylation of EFNB1’s PBM disrupts the EFNB1-EGFR association.

## Graphical Abstract

**Figure:**
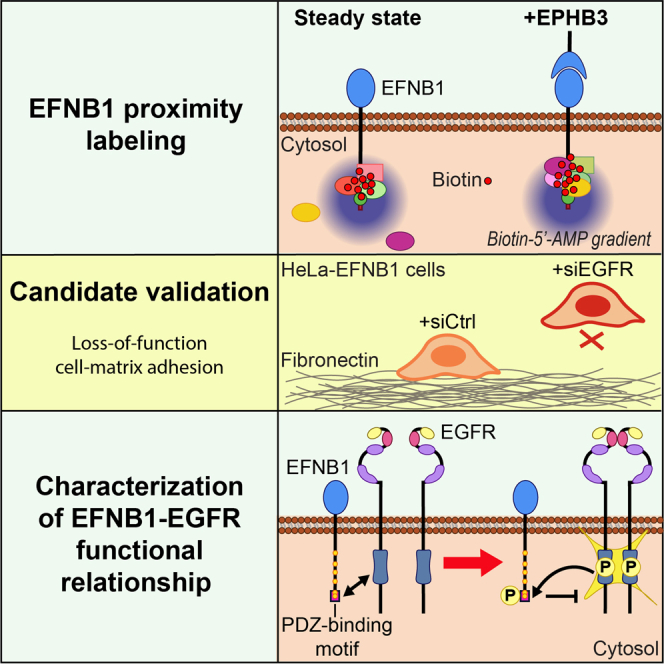

## In Brief

Osornio-Hernández *et al*. delineate the proximity network of EFNB1 and identify EGFR as a key proximity partner required for EFNB1-dependent cell adhesion to fibronectin. They show that EFNB1 associates *via* its PDZ-binding motif (PBM) with EGFR, which can phosphorylate this PBM on two tyrosine residues. This phosphorylation results in the disruption of the EFNB1–EGFR association and a loss of cell-matrix adhesion, thus revealing that EGFR regulates EFNB1 function.

The Erythropoietin-Producing Hepatocellular receptors (EPH) represent the most extensive subfamily of receptors tyrosine kinase (RTKs) in humans (1). This subfamily of receptors comprises 14 members that are classified as EPHA (EPHA1-8,10) or EPHB (EPHB1-4,6) based on sequence homology and ligand-binding preferences (2). Importantly, EPH receptors differ from other RTKs in that their ligands, the ephrins, are membrane-bound proteins. Therefore, EPH receptors and ephrins are commonly implicated in short-range cell-cell communication (3, 4). Ephrins can be tethered to the plasma membrane by a GPI anchor (ephrin-A1-5) or display a transmembrane domain (ephrin-B1-3) (5). Interestingly, one particularity of the EPH/ephrin system is that when EPH receptors and ephrins interact at apposed cells, both proteins may act as a receptor and ligand at the same time, a process known as bidirectional signaling (6). Signaling downstream of EPH receptors is referred as being “forward” while the one downstream of ephrins is called “reverse” (2).

The three ephrin-Bs encoded in the human genome display an extracellular EPH-receptor binding domain, a single transmembrane domain, and a short intracellular domain of ∼80 aa. The latter includes five Tyr residues that are conserved within the three human ephrin-Bs and across evolution (7). The last four amino acids (YYKV) at the C-terminus constitute a PDZ-binding motif (PBM), which also includes the last two of the conserved Tyr residues (8). Reverse signaling downstream of ephrin-Bs is mainly classified as pTyr-dependent or PDZ-dependent, although other signaling mechanisms have been also described (9). In the case of pTyr-dependent signaling, EPH receptors binding to ephrin-Bs leads to the phosphorylation of Tyr residues in ephrin-Bs’ cytoplasmic tails. This phosphorylation is mostly mediated by SRC family kinases (10, 11). The resulting pTyr residues are recognized by cytoplasmic proteins bearing SH2 or PTB domains to initiate the nucleation of signaling complexes. Ephrin-Bs intracellular effectors that act *via* this mechanism include NCK2 and STAT3 (12, 13). In addition, ephrin-Bs nucleate signaling complexes by recruiting PDZ-domain proteins through their PBMs (14). Effectors binding in this way include PTPN13 and PDZ-RGS3 (11, 15). Moreover, ephrin-Bs can associate with signaling effectors independent of pTyr residues or PDZ-dependent interactions, as observed for PAR6 (16). Remarkably, Tyr phosphorylation in the intracellular domain of ephrin-Bs not only facilitates the formation of signaling complexes, but can also trigger their disassembly, as for PAR6 and dishevelled (DVL) (16, 17). Together, EPH receptors and ephrins control multiple cellular processes, including tissue patterning, axon pathfinding, tissue boundary formation, cell adhesion and migration (6, 18). Notably, ephrin-Bs are known to play key roles in the regulation of cell-cell adhesion (16, 19) and cell-matrix adhesion (20). In particular, reverse signaling downstream of ephrin-Bs is known to enhance integrin-dependent cell adhesion to ECM proteins (21, 22, 23). Despite our current knowledge on reverse signaling, the molecular mechanisms underlying multiple ephrin-B-dependent functions and phenotypes are not completely understood, suggesting that several ephrin-B intracellular effectors are yet to be identified. Previous efforts to identify ephrin-Bs intracellular effectors were conducted primarily by AP-MS (24) and yeast two-hybrid screenings (12, 15, 25, 26). Since these techniques favor the identification of more stable interactions, intracellular effectors that associate transiently with ephrin-Bs are most likely underrepresented in the literature. Moreover, studying protein–protein interactions (PPIs) by AP-MS requires solubilization of protein complexes, which may be challenging for plasma membrane proteins such as ephrin-Bs. To overcome these limitations, proximity labeling proteomics techniques have been developed (27, 28). These techniques employ enzymes such as peroxidases (*e.g.* APEX) or biotin ligases (*e.g.* BirA, TurboID, miniTurbo (miniT) or UltraID), which vary in their ability to generate reactive biotin radicals. When fused to a protein of interest, these enzymes will allow the labeling of neighboring proteins. The resulting biotinylated proteins are then affinity purified using streptavidin and identified by MS (29, 30, 31, 32). Here, we employed a proteomics approach using the miniT biotin ligase to delineate the EFNB1 proximity network under steady state and EPHB3-stimulation conditions. With this approach, we identified 104 EFNB1 proximity partners, from which we could distinguish three main groups: EPH receptor stimulation-dependent, stimulation-independent and negatively modulated by EPH receptor stimulation. We further characterized the functional relationship between EFNB1 and one of its proximity partners, the epidermal growth factor receptor (EGFR). While the association between EFNB1 and EGFR was previously suspected (33), several aspects of this relationship remain to be elucidated. In this study, we established that the EFNB1-EGFR complex formation is dependent on the PDZ-binding motif (PBM) of EFNB1. Notably, we found that EGFR directly phosphorylates tyrosine residues located in EFNB1’s PBM, leading to dissociation of the EFNB1–EGFR complex. Additionally, our data indicate that this interaction is essential for EFNB1-mediated cell adhesion to fibronectin. Collectively, these findings reveal a functional interplay between EFNB1 and EGFR.

## Experimental Procedures

### Cell Culture and Transfections

Human embryonic kidney 293T cells (HEK293T), HEK293 T-Rex, and HeLa T-REx cell lines were grown at 37 °C under 5% CO_2_ in Dulbecco’s Modified Eagle’s medium (DMEM, Multicell, Wisent) supplemented with 4.5 g/L glucose and 10% fetal bovine serum (FBS, Gibco). T-REx stable cell lines were generated using the T-REx system by Invitrogen. Briefly, parental Flp-In T-REx cell lines were co-transfected with pcDNA5/FRT/TO containing the cDNA of interest and the pOG44 plasmid (Flp recombinase), using Lipofectamine 2000 (Thermo Fisher Scientific). Selection and maintenance of stable cell lines was achieved by adding hygromycin-B (BioShop) to the culture media (100 μg/ml for HEK293 and 300 μg/ml for HeLa). Expression of the proteins of interest in these cell lines was induced by supplementing the culture media with 1 μg/ml tetracycline (Wisent) for 48 h. For biotinylation experiments, cells were grown in biotin-depleted media for at least 72 h. To achieve this, the culture media was complemented with biotin-depleted FBS, which was obtained by incubating FBS with streptavidin-beads (Sigma-Aldrich) (5 μl of packed bead volume per 5 ml of FBS) for 3 h at 4 °C and sterilized by filtration through a 0.22 μm filter. Time-resolved proximity biotinylation was achieved by adding 50 μM biotin (Sigma-Aldrich) to miniT-expressing cells for 20 min at 37 °C. Transient transfection of HEK293T cells was done using polyethylenimine (PEI) and cells were allowed to express constructs for at least 36 h following the transfection. Lipofectamine RNAiMAX (Thermo Fisher Scientific) was used to transfect siRNAs, and depletion of target proteins was evaluated 48 h post-transfection.

### Plasmids and Constructs

For proximity biotinylation experiments and stable expression in mammalian cells, the human sequence of EFNB1 and the YFP sequence were subcloned into pcDNA5/FRT/TO-miniT-Flag (34). The human sequences of EFNB1, EPHB3 or EPHB3-Flag were subcloned into pcDNA5/FRT/TO. For C-terminal GFP tagged EFNB1 constructs, the human sequence of EFNB1 was subcloned into pcDNA5/FRT/TO-GFP. Deletion mutants and point mutations of EFNB1 were generated using the Q5 Site-Directed Mutagenesis Kit (New England Biolabs) following the instructions provided by the manufacturer. EFNB1-6F Y/F mutant was obtained by assembly of double-stranded DNA fragments (Integrated DNA Technologies) using the HiFi DNA assembly kit (New England Biolabs). Human EGFR was overexpressed from the pcDNA3.1 vector and was a gift from Thomas Moss, Université Laval. For bacteria expression, the human sequence of EFNB1 intracellular domain (aa259–346) was subcloned into the pMAL-c5X vector and was tagged in the N-terminus with the maltose-binding protein (MBP). Target sequences of all siRNAs used in this study are provided as supplementary information (Supplemental Table S1). All constructs were validated by Sanger sequencing.

### Cell Lysis and Affinity Purification

Cells used in proximity labeling experiments were lysed as described (34). Briefly, four 15 cm dishes per condition were lysed in cold RIPA buffer (50 mM Tris-HCl pH 7.5, 150 mM NaCl, 1 mM EGTA, 1 mM EDTA, 1% Triton-X100, 0.1% SDS, 0.5% sodium deoxycholate) complemented with protease inhibitors (1 mM PMSF, 10 μg/ml aprotinin, 10 μg/ml leupeptin and 10 μg/ml pepstatin). Cell lysates were agitated at 4 °C for 1 h with 125 units of benzonase (Sigma-Aldrich), followed by sonication and centrifugation at 21,000*g* for 30 min at 4 °C. Cell supernatants were then incubated with 40 μl of packed streptavidin-coupled beads (Sigma-Aldrich) for 3 h at 4 °C. Beads were washed twice with 1 ml of lysis buffer, twice with 1 ml of KLB buffer (20 mM Tris-HCl pH 7.4, 150 mM NaCl, 1 mM EDTA, 1% IGEPAL, 0.5% sodium deoxycholate, 10 mM beta-glycerophosphate, 50 mM NaF, 10 mM sodium pyrophosphate, 10% glycerol) and twice with 1 ml of 20 mM Tris-HCl, pH 7.4. HeLa T-REx and HEK293T cells used for immunoprecipitation (GFP or Flag) or pulldowns were lysed in cold KLB buffer complemented with a protease inhibitor cocktail (Sigma-Aldrich, P8340) and phosphatase inhibitors. Cell lysates were incubated for 15 min on ice and centrifuged at 21,000*g* for 20 min at 4 °C. Protein concentrations were measured with a BCA kit (Thermo Fisher Scientific). Cleared supernatants were then agitated at 4 °C with 10 μl of packed GFP-trap beads or anti-Flag M2 affinity gel (Sigma-Aldrich) for 1.5 h or 2 h respectively. Beads were washed three times (5 min each) with 1 ml of lysis buffer and boiled with 10 uL of 4X Laemmli buffer for 4 min.

### Western Blotting and Antibodies

Cell lysates and affinity-purified samples were resolved using 8% or 10% polyacrylamide gels and transferred to nitrocellulose membranes (Bio-Rad). Membranes were blocked in 5% milk and incubated with antibodies diluted in 1% milk in TBS. Antibodies utilized are the following: anti-Flag M2-HRP (Sigma, A8592), anti-EFNB1 (R&D systems, AF473), anti-GFP (Invitrogen, A11122), 27B10-HRP (Cytoskeleton, APY03-HRP-S), anti-goat-HRP (Thermo Fisher Scientific, 31,402), and anti-pEFNB (Cusabio, CSB-PA007934). Antibodies purchased from Cell Signaling Technology include anti-actin (#3700), anti-EGFR (#4267), anti-pEGFR (#3777), anti-mouse-HRP (#7076), and anti-rabbit-HRP (#7074). For detection of biotinylated proteins using streptavidin-HRP (Invitrogen, 43–4323), membranes were blocked in 1% BSA, 0.2% Triton-X100 in PBS. Incubation with streptavidin-HRP was carried out in blocking buffer for 40 min, followed by 5 min incubation in 10% BSA, 1% Triton-X100 in PBS and three washes (10 min each) in PBS. Detection of signals from HRP-conjugated antibodies were developed using Clarity Western ECL Substrate (Bio-Rad) and the Amersham Imager 600 (GE Healthcare).

### Immunofluorescence

Expression of miniT fusions in HEK293 T-REx cells or EFNB1 constructs in HeLa T-REx cells was induced for 48 h before plating on fibronectin-coated plates (Ibidi, 80366). Prior to plating, cells were resuspended using cell dissociation buffer (Gibco) and DMEM supplemented with 10% FBS. Cells were allowed to adhere to the bottom of the dish for 3 h at 37 °C under 5% CO_2_. After this time, cells were fixed with 4% PFA (BioShop) and permeabilized using 0.2% Triton-X100 (Sigma) in PBS (15 min at RT each). Blocking was carried out for 1 h at RT in 0.1% Triton-X100, 10% goat or donkey serum (Wisent) in PBS. Incubation with mouse anti-Flag M2 (Sigma, F1804) or goat anti-EFNB1 (R&D systems, AF473) was done in blocking buffer overnight at 4 °C with gentle agitation. Unbound antibody was removed by three washes with 0.1% Triton-X100 in PBS (10 min each). Incubation with Alexa-488-conjugated anti-mouse (Cell Signaling Technologies, #4408) or anti-goat (Thermo Fisher, #A-11055) antibody was performed for 1 h at RT in 0.1% IGEPAL in PBS. Unbound secondary antibody was washed three times (10 min each) in the same solution and DAPI was added to the first wash. Finally, cells were rinsed twice in PBS and imaged using an Olympus FV1000 confocal microscope with an Olympus PLAPON 60× oil 1.42 NA objective at 1.5X numerical zoom (miniT fusions) or with an Olympus PlanApo 0.90 NA objective at 2X numerical zoom (GFP-tagged and untagged EFNB1 constructs). For GFP-tagged EFNB1 fusions, HEK293T cells were plated on fibronectin-coated dishes (Ibidi, 81156) 24 h prior transfection with plasmids encoding EFNB1-GFP fusions. 36 h post-transfection, cells were fixed with 4% PFA (BioShop) and stained for 10 min with DAPI in PBS. DAPI excess was removed by washing 3 times with PBS (10 min each) and cells were imaged using the GFP signal and an Olympus FV1000 confocal microscope at 40×, as described above.

### Protein Expression and Purification

Expression of MBP-tagged constructs was induced in BL21 (DE3) *E. coli* with 0.5 mM IPTG for 18 h at 16 °C. Bacteria expressing MBP-tagged constructs were pelleted by centrifugation and subsequentially resuspended and sonicated in the following buffer: 20 mM Tris-HCl pH 7.4, 200 mM NaCl, 1 mM EDTA, 1 mM DTT, and 1 mM PMSF complemented with a cocktail of protease inhibitors (Roche). Cell lysates were centrifugated at 24,000*g* for 20 min at 4 °C, and supernatants were agitated with pre-washed amylose beads (New England Biolabs) for 1.5 h at RT. Beads were then washed three times with lysis buffer (10 min each at RT). For kinase assays, the MBP-fusion proteins were eluted from the amylose beads by agitating them three times with lysis buffer supplemented with 10 mM maltose (10 min each at RT). For pulldown assays, MBP–fusion proteins were kept bound to the beads.

### Pulldowns

Lysates of HEK293T cells overexpressing EGFR were pre-cleared with amylose beads bound to MBP for 2 h at 4 °C with agitation (10 μg of MBP per mL of lysate). Beads were discarded, and the cleared lysates were agitated with 10 μg of MBP-B1-ICD fusions bound to amylose beads for 2 h at 4 °C. Beads were then washed three times for 5 min with washing buffer (20 mM Tris-HCl pH 8, 200 mM NaCl, 1 mM EDTA, 0.5% IGEPAL) supplemented with protease inhibitors (Sigma-Aldrich, P8340). Finally, beads were boiled for 4 min in 4X Laemmli buffer.

### Peptide Arrays

Peptides including the six Tyr residues present in the intracellular domain of EFNB1 were synthesized as 13-mers with the Tyr of interest in the middle position. Non-phosphorylable controls were generated by replacing the Tyr residues by Phe. Peptide array synthesis was performed as previously described by Dionne *et al*. (35). Briefly, peptides were synthesized on cellulose membranes using Fmoc chemistry and an automated MultiPep peptide synthesizer with SPOT synthesis option (CEM corporation). Peptide sequences and positions on membranes can be found in Supplemental Table S2.

### *In Vitro* Radioactive Kinase Assays

Kinase reactions were set up on ice using 1 μg of recombinant human EGFR catalytic domain (aa 668–1210) fused to GST (Thermo Fisher, PR7295B) and 3 μg of MBP-EFNB1-ICD fusions in a kinase reaction buffer (25 mM Tris-HCl pH 7.5, 10 mM MgCl_2_, 0.5 mM EGTA, 0.5 mM Na_3_VO_4_, 5 mM β-glycerophosphate, 2.5 mM DTT, 0.01% Triton X-100). To start the reaction, cold ATP to a final concentration of 200 μM and 5 μCi [*γ-*^*32*^*P*]-ATP (PerkinElmer (now Revvity), BLU502Z250UC) were added to the reaction mix, which was incubated at 37 °C for 1 h. The reaction was stopped by adding Laemmli buffer and boiling for 4 min. The mix was then resolved in a 10% polyacrylamide gel, which was subsequentially stained with Coomassie Brilliant Blue (BioBasic) and dried using a gel drier (Bio-Rad). For peptide arrays, membranes were hydrated in 100% MeOH and rinsed sequentially in TBS-T, TBS and kinase reaction buffer (5 min each). The arrays were then incubated with 10 μg of EGFR catalytic domain, 40 μM cold ATP and 50 μCi [*γ-*^*32*^*P*]-ATP in kinase reaction buffer for 1 h at 37 °C. The reaction was stopped with three washes (20 min each) in stopping buffer (8 M urea, 1% SDS, 0.5% beta-mercaptoethanol), followed by one wash of 20 min with 50% ethanol, 10% acetic acid. Finally, the array was rinsed with 95% ethanol and dried. Once dried (gel or array), incorporation of [*γ-*^*32*^*P*]-ATP was assessed by autoradiography using an Amersham Typhoon apparatus (GE Healthcare). The Protein Array Analyzer macro for Image J (Gilles Carpentier) was used for signal quantification.

### Cell Attachment Assay

The cell attachment assay was adapted from the one described by Huynh-Do *et al*. and Meyer *et al*. (21, 22, 23). Briefly, 48-well plates were incubated overnight at 4 °C with 5 μg/ml fibronectin (Sigma-Aldrich) in PBS. After this time, wells were rinsed twice with PBS and blocked with 1% BSA in PBS for 10 min at 37 °C. The blocking solution was removed by washing twice with PBS and the plates were either stored at 4 °C with PBS or filled with serum-free DMEM. Cells used in the assay were serum-starved for at least 18 h and resuspended in serum-free DMEM using cell dissociation buffer. Cells were then plated on the fibronectin coated plates at a density of 50,000 cells per well and incubated for 15 min at 37 °C. After this time, unattached cells were dislodged by three brisk taps on the table and removed by PBS washing. Attached cells were fixed with 1% glutaraldehyde in PBS (10 min at RT), stained with 0.1% crystal violet in PBS (1 h at RT) and quantified by eluting the crystal violet from the cells using 10% acetic acid and measuring the absorbance at 590 nm.

### Receptor Stimulation and Cell Overlay

Experiments where EFNB1 expressing cells were stimulated with EPHB3 were done by overlaying a cell suspension of EPHB3-expressing cells on top of a monolayer of EFNB1-expressing cells. Briefly, the 2 cell populations were grown to a fully confluent monolayer before the cell resuspension and overlay, aiming to achieve a 1:1 cell ratio during the stimulation. EFNB1-cells and EPHB3-cells were serum-starved using plain DMEM supplemented with 1 μg/ml tetracycline, 18 h prior to mixing. Following starvation, EPHB3-cells were detached using cell dissociation buffer and resuspended in serum-free DMEM (10 ml for 15 cm dishes or 5 ml for 10 cm dishes) using cell dissociation buffer (Gibco). Culture media was removed from the monolayer of EFNB1-cells before adding the EPHB3-cell suspension on top and incubating for 20 min at 37 °C. For proximity labeling experiments, the EPHB3-cell suspension was supplemented with 50 μM biotin to couple the stimulation with the biotinylation of proximal proteins. After cell mixing, cells were rinsed twice with cold PBS prior to cell lysis. For the “no stimulation” conditions, EPHB3-cells were replaced by parental HEK293 T-Rex cells.

For experiments in which EPHB3-expressing cells were stimulated with EFNB1 or EFNB1 deletion mutants, we followed the same protocol described above, with the modification that a monolayer of EPHB3-expressing cells was stimulated with a suspension of cells expressing full-length EFNB1 or the respective deletion mutants.

### Gene Ontology Enrichment Analysis

Gene ontology enrichment analysis was performed using PANTHER (36, 37) (overrepresentation test analysis, Fisher test type, FDR correction). Graphs representing the -Log(*p*-value) of the 10 most significative enriched terms for biological process, cellular component or molecular function were generated using GraphPad Prism.

### Experimental Design and Statistical Rationale

MiniT experiments were performed in biological triplicates (n = 3), which were required and sufficient to filter out background from *bona fide* proximity partners using the YFP-miniT-Flag condition as a control through SAINT analysis. Affinity purification experiments were also performed in biological triplicates (n = 3), employing cells from successive passages on different days. *In vitro* kinase assays were performed in triplicates (n = 3) done on different days. Attachment assays were performed in biological triplicates (n = 3) and each biological replicate was plated in technical triplicates. The percentage of attachment relative to the positive control was calculated for each technical replicate and the average of the three technical replicates was considered the value of the biological replicate. Statistical significance was then assessed by one-way ANOVA and Dunnett’s post hoc test using the values of three biological replicates and GraphPad Prism. In all cases, controls for each experiment were processed simultaneously with the experimental samples.

### Mass Spectrometry

Following streptavidin affinity purification, biotinylated proteins were eluted from the streptavidin-coupled beads by agitating the beads 3 times (10 min each at RT) with 150 μl of 50 mM H_3_PO_4_, pH 2. Eluted proteins were then trypsin digested on a strong cation exchange (SCX) chromatography medium (polysulfoethyl A, particle size 12 μm, pore size 300 Å, Western Analytical) in a spin tip as described (38). Briefly, conditioned SCX resin (2–3 μl) was packed in a 10 μl pipette tip with 0.6 μl bed of SCX fixed at its end (ZipTip, Millipore). The column was then washed five times with 60 μl of 10 mM KH_2_PO_4_, pH 3 (centrifugation at 100*g* for 3 min). The phosphoric acid elution containing biotinylated proteins was then loaded onto the column by centrifuging 60 μl of sample at 100g for 3 min until all sample was loaded. The column was then washed with 60 μl of 10 mM KH_2_PO_4_, pH 3, followed by one wash with 30 μl of HPLC-grade water. After this, 3 μl of 100 mM TCEP (in 100 mM Tris-HCl, pH 9) were loaded in the column and incubated at RT for 30 min. TCEP was then washed with 60 μl of HPLC-grade water. Next, 2 μl of Sequencing Grade Modified Trypsin (Promega) (2 mg/ml in 100 mM Tris-HCl, pH 8, 10 mM iodoacetamide) were loaded in the column and incubated for 2 h at RT. Digested peptides were then eluted 4 times with 5 μl of 200 mM NH_4_HCO_3_, pH 8. The elution containing the digested peptides was then desalted by stage tip as described (39). Briefly, stage tips were prepared by inserting two disks of C-18 extraction disks (Thermo Fisher, 143862) into a 10 μl pipette tip. Columns were then conditioned by passing 30 μl of methanol thought the stage tip (centrifugation at 1000*g* for 3.5 min), followed by 20 μl of buffer A (3% acetonitrile, 1% TFA, 0.5% acetic acid). The elution containing the digested peptides was acidified with 40 μl of buffer A before passing it through the column. After this, the column was washed with 30 μl of buffer A. Peptides were then eluted two times with 22.5 μl of buffer B (0.5% acetic acid, 80% acetonitrile) and dried in a SpeedVac vacuum concentrator (2 h at 45 °C, V-AL). Dried peptides were then resuspended and separated by liquid chromatography as described (34). Briefly, dried peptides were resuspended in 15 μl of loading solvent (2% acetonitrile, 0.05% TFA), and 5 μl of the solution were injected onto a C18 Pepmap cartridge precolumn (300 μm inner diameter × 5 mm; Dionex/Thermo Fisher Scientific) at a flow rate of 20 μl/min using the same loading solvent. Following 5 min desalting, the precolumn was switched in-line with an analytical separation column (75 μm inner diameter × 50 cm) packed with 3 μm ReproSil-Pur C18-AQ resin (Dr Maisch HPLC GmbH), pre-equilibrated in 95% solvent A (2% acetonitrile, 0.1% formic acid), and 5% solvent B (80% acetonitrile, 0.1% formic acid). Peptide separation was carried out using a 90 min gradient from 5% to 40% solvent B at a flow rate of 300 nl/min generated by an UltiMate 3000 RSLCnano system (Dionex/Thermo Fisher Scientific). Eluted peptides were analyzed using an Orbitrap Fusion mass spectrometer equipped with a nanoelectrospray ion source (Thermo Fisher Scientific). Identification of biotinylated proteins was carried as previously described (40). Briefly, the XCalibur software version 4.3.73.11 (Thermo Scientific) was used for data-dependent acquisition of mass spectra. Acquisition of full scan mass spectra (350–1800 m/z) was performed in the orbitrap using an automatic gain control (AGC) target of 4 × 10^5^ and a maximum injection time of 50 ms at a resolution of 120,000. The quadrupole analyzer enabled the isolation of ions in a window of 1.6 m/z and fragmentation by Higher energy Collision-induced Dissociation (HCD) with 35% energy. The linear ion trap at a rapid scan rate was then used to detect the resulting fragments with a maximum injection time of 50 ms and an AGC target of 1 × 10^4^. A period of 20 s and a tolerance of 10 ppm was set for dynamic exclusion of previously fragmented peptides. The Thermo Proteome Discoverer version 2.5.0.400 (Thermo Scientific) was used to generate all MS/MS peak lists. The Mascot search engine (Matrix Science; version 2.8.0) was used to analyze the MGF sample files using the Uniprot *Homo sapiens* reference proteome (September 2022, 79,759 entries) complemented with common contaminants from the Global Proteome Machine (GPM, September 2020) and the YFP and miniT-Flag sequences assuming tryptic digestion. Searches were carried out with a parent ion tolerance of 10 ppm and a fragment ion mass tolerance of 0.60 Da. Accepted variable modifications were oxidation of methionine, deamidation of asparagine and glutamine, and phosphorylation of serine, threonine and tyrosine. Fixed modifications included carbamidomethyl of cysteine. A maximum of two missing cleavages were allowed. MS/MS-based peptide and protein identifications were then validated using the Scaffold software version 5.0.1 (Proteome Software Inc., Portland, OR). Peptide identifications were accepted if they could be established at greater than 91% probability to achieve an FDR less than 1% by the Scaffold Local FDR algorithm. Protein identifications were accepted if they could be established at greater than 63% probability to achieve an FDR less than 1% and contained at least 2 identified peptides. Protein probabilities were assigned by the Protein Prophet algorithm (41). Proteins that contained similar peptides and could not be differentiated based on MS/MS analysis alone were grouped to satisfy the principles of parsimony. To distinguish *bona fide* proximity partners from background contaminants and non-specific interactions, MS data were analyzed with SAINTexpress (42) *via* the Resource for Evaluation of Protein Interaction Networks (REPRINT) online tool using default parameters and MS data from the YFP-miniT-Flag control. Proteins with a BFDR ≤1% were considered true proximity partners. Finally, interaction networks were generated with Cytoscape software version 3.9.0 (43) and dot plots with the ProHits-viz online tool (44).

## Results

### EFNB1 Proximity Labeling Proteomics Identifies EPHB3 Receptor Stimulation-Dependent and Independent Proximity Partners

**Fig. 1:**
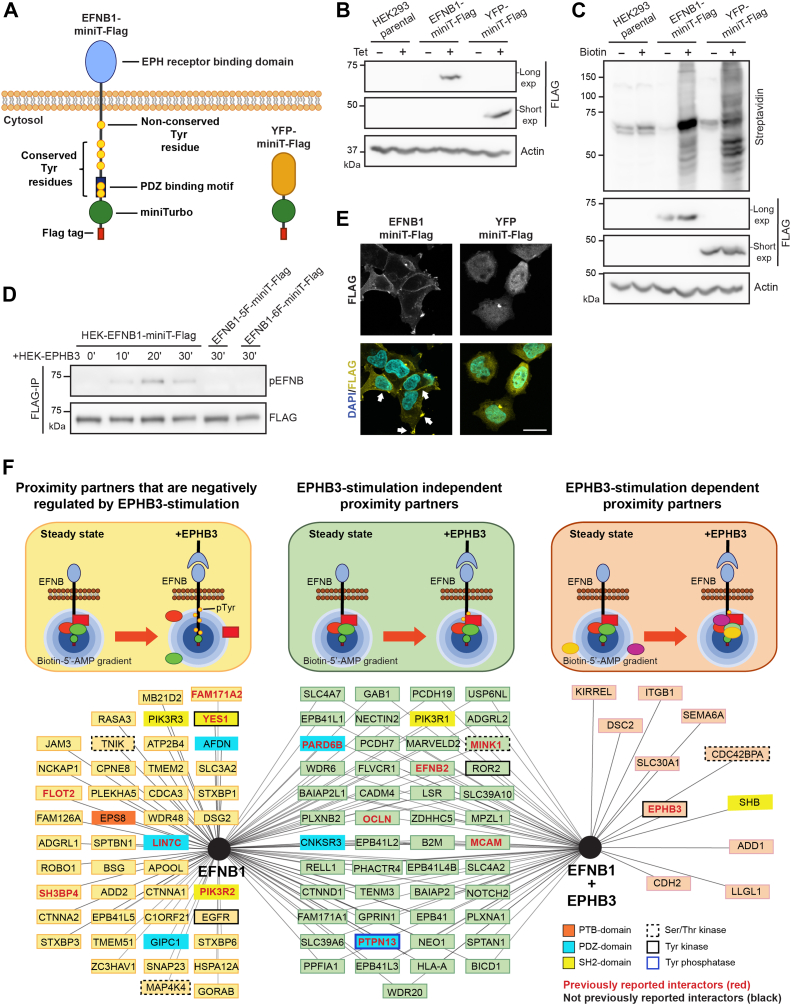
**Proximity labeling proteomics reveals EFNB1’s steady state and EPHB3 stimulation-dependent networks.***A*, schematic representation of miniT-Flag fusions. The promiscuous biotin ligase miniT and a Flag tag were fused at the C-terminus of EFNB1 or YFP (control). *B*, expression of miniT-Flag constructs in HEK293 T-REx stable cell lines. Tetracycline (Tet) treatment induces expression of EFNB1-miniT-Flag or YFP-miniT-Flag fusions in HEK293 T-REx stable cell lines. *C*, biotinylation of proximal proteins by miniT-Flag fusions was assessed by western blotting using streptavidin-HRP. Addition of biotin to Tet-treated EFNB1-miniT-Flag or YFP-miniT-Flag stable cells lines leads to biotinylation of endogenous proteins. *D*, EFNB1-miniT-Flag expressing cells (HEK-EFNB1-miniT-Flag) were stimulated with EPHB3 expressing cells (HEK-EPHB3) for different times. EFNB1-miniT-Flag Tyr phosphorylation was assessed with an anti-phospho-EFNB antibody (pEFNB). Y/F mutants of EFNB1 (EFNB1-5F-miniT-Flag and EFNB1-6F-miniT-Flag) were used as negative controls. EFNB1-5F-miniT-Flag carries Y/F mutations at positions 317, 324, 329, 343 and 344. EFNB1-6F-miniT-Flag carries Y/F mutations at positions 313, 317, 324, 329, 343 and 344. *E*, subcellular localization of miniT-Flag fusions was analyzed by immunofluorescence using anti-Flag antibodies. EFNB1-miniT is enriched at the plasma membrane (*white arrows*) while YFP-miniT is distributed throughout the cells. Scale bar represents 20 *μ*m. *F*, proximity network for EFNB1 in steady state and under EPHB3-stimulation (EFNB1+EPHB3). The network is divided into three main sections: (*left*) proximity partners that are negatively regulated by EPHB3-stimulation; (*center*) EPHB3-stimulation independent proximity partners and (*right*) EPHB3-stimulation dependent proximity partners. Only proteins identified with high confidence (Bayesian False Discovery Rate *BFDR* ≤1%) are displayed.

To identify EFNB1 proximity partners, we established independent HEK293 T-REx cell lines stably expressing EFNB1 or YFP fused at their C-terminus to the promiscuous biotin ligase miniTurbo (EFNB1-miniT-Flag and YFP-miniT-Flag) (Fig. 1*A*). We validated that the addition of tetracycline to the culture media induced the expression of the miniT fusions (Fig. 1*B*). We corroborated that miniT fusions are active biotin ligases by adding biotin to cell media for 20 min, which led to biotinylation of endogenous proteins across a range of molecular weights (Fig. 1*C*). To confirm that EFNB1-miniT-Flag can still be Tyr phosphorylated following EPHB3 stimulation, we overlayed a suspension of EPHB3-expressing cells (HEK-EPHB3) on top of a monolayer of EFNB1-miniT-Flag expressing cells for 0 to 30 min. EFNB1 affinity purification followed by anti-pTyr Western blotting showed that EFNB1-miniT-Flag Tyr phosphorylation is maximal at 20 min of stimulation (Fig. 1*D*). Importantly, no phosphorylation was detected in non-phosphorylable EFNB1 Y/F mutants, in which the five conserved (5F) or all six (6F) Tyr residues were replaced by Phe. Finally, as the addition of miniT to the intracellular domain of EFNB1 could potentially cause its mislocalization, we assessed the subcellular localization of EFNB1-miniT-Flag and YFP-miniT-Flag by immunofluorescence. We found that only EFNB1-miniT-Flag was enriched at the plasma membrane, as expected (Fig. 1*E*).

To identify proteins that are recruited proximal to EFNB1 following EPHB3 stimulation, we coupled biotinylation with cell overlay. We exposed a monolayer of EFNB1-miniT-Flag expressing cells with a suspension of EPHB3-expressing cells in the presence of 50 μM biotin. We selected a stimulation time of 20 min based on our previous finding showing that EFNB1 Tyr phosphorylation reaches its maximum at this time (Fig. 1*D*). We used as a control a suspension of parental HEK293 T-REx cells, which express low levels of EPH receptors (45) and therefore, do not induce EFNB1 Tyr phosphorylation (Fig. 1*D*). Biotinylated proteins were affinity-purified using streptavidin-coupled beads and identified by mass spectrometry. We performed three biological replicates and removed unspecific background using the SAINT algorithm and MS data from the YFP-miniT-Flag control (46, 47). Proteins identified with high confidence (*Bayesian False Discovery Rate* (*BFDR*) ≤1%) were then classified as EPHB3-stimulation dependent (11 candidates), EPHB3-stimulation independent (49 proteins), or proximity partners negatively regulated by EPHB3 receptor stimulation (44 proteins) (Fig. 1*F* and Supplemental Fig. S1, and supplemental Tables S3–S5). Notably, according to the existing literature and the BioGRID database (48), ∼86% of the identified proximity partners (90 proteins) were not previously reported to associate with EFNB1. We also found 14 candidates that were previously linked to EFNB1 signaling, including SRC family Tyr kinase YES1, which was previously reported to phosphorylate ephrin-Bs (11, 49); Tyr phosphatase PTPN13, which binds to ephrin-Bs in a PDZ domain-dependent manner and is able to dephosphorylate ephrin-Bs intracellular domain (11); Par polarity complex member PARD6B (16); and finally lipid raft protein FLOT2 (50). Moreover, in a large-scale study using affinity purification followed by MS (51), a number of candidates that we identified were also found: SH3BP4, LIN7C, FAM171A2, PIK3R2, EFNB2, and MINK1. Likewise, EFNB1 was identified in proximity labeling proteomics analyses of OCLN (52) and MCAM (53). Further supporting the validity of our approach, gene ontology (GO) analyses highlighted an enrichment for EFNB1 proximity partners in processes such as cell adhesion, cell–cell adhesion, cell migration, and regulation of the actin cytoskeleton (Supplemental Fig. S2*A*), all of which previously reported as being regulated by EFNB reverse signaling (2, 3, 5, 6, 18). Furthermore, the GO analysis for cellular compartment (Supplemental Fig. S2*B*), did not only highlight plasma membrane, but also cell-cell junctions, cell-substrate junctions and focal adhesions, all of which are in line with established functions for EFNB1 in the regulation of tight and gap junctions, integrin-dependent ECM attachment and focal adhesions (12, 16, 19, 22, 23, 54). Together, these results support the validity of our EFNB1 proximity labeling approach.

### EGFR Associates With EFNB1 and is Required for EFNB1-Dependent Cell Adhesion to Fibronectin

**Fig. 2:**
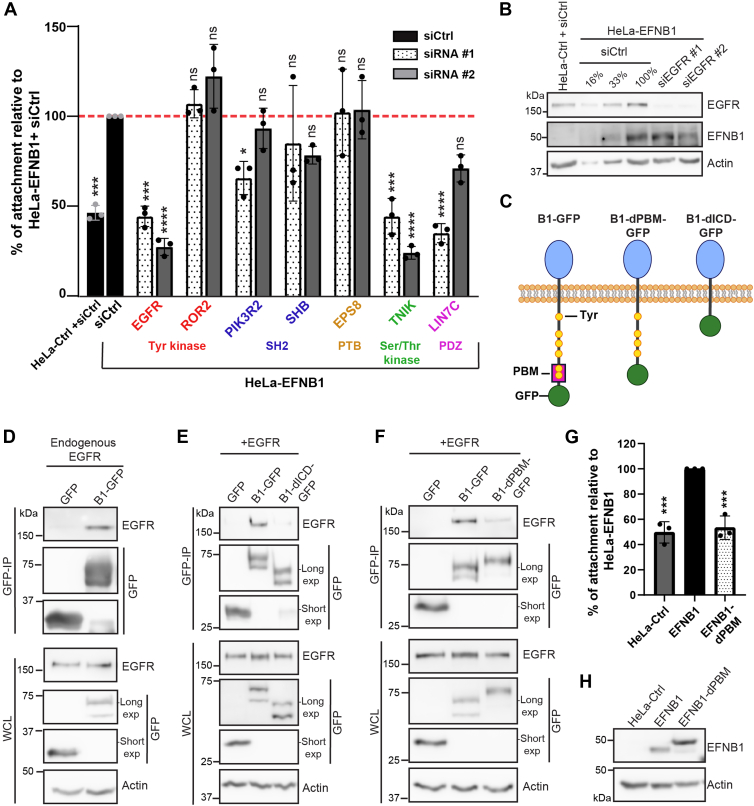
**EGFR associates with EFNB1 and is required for EFNB1-dependent cell adhesion to fibronectin.***A*, HeLa cells overexpressing EFNB1 (HeLa-EFNB1) were transfected independently with two siRNAs to evaluate the effect of targets’ depletion on EFNB1-dependent cell adhesion to fibronectin. Values were normalized relative to HeLa-EFNB1 cells treated with siCtrl (*dashed red line*). Error bars indicate SD (∗*p* < 0.05, ∗∗∗*p* < 0.0005, ∗∗∗∗*p* < 0.0001; one-way ANOVA and Dunnett’s post-hoc test). *B*, Western blot analysis of EGFR and EFNB1 levels from HeLa-Ctrl or HeLa-EFNB1 cells treated with siCtrl or siEGFR (from *A*). Numbers (in %) represent a fraction of loading. *C*, schematic representation of GFP-tagged EFNB1 fusions; mutants lacking the PBM (aa343–346) (B1-dPBM-GFP) or deletion mutant lacking most of the intracellular domain (aa 269–346) (B1-dICD-GFP). *D*, Western blot analysis of endogenous EGFR following GFP-immunoprecipitation from HEK293T cells overexpressing B1-GFP or GFP alone. *E* and *F*, Western blot analysis of EGFR following GFP-immunoprecipitation using cell lysates of HEK293T cells overexpressing EGFR and EFNB1 GFP-tagged constructs. *G*, cell adhesion to fibronectin was evaluated for HeLa T-REx cell lines stably overexpressing untagged WT EFNB1 or deletion mutants lacking the PBM (EFNB1-dPBM). Values were normalized relative to HeLa-EFNB1 cells. Error bars indicate SD (∗∗∗*p* < 0.001; one-way ANOVA and Dunnett’s post-hoc test). *H*, EFNB1 Western blot from HeLa T-REx lysates used in *panel G*.

To test a possible functional relationship between EFNB1 and its miniTurbo-identified proximity partners *via* a loss-of-function approach, we adapted a previously reported cell-matrix attachment assay (21, 22, 23). We generated HeLa T-REx cell lines stably expressing EFNB1 (HeLa-EFNB1) and a HeLa-Ctrl cell line. Following 15 min plating on fibronectin coated dishes, cells still in suspension were removed by washing, and attached cells were quantified by Crystal violet staining. As expected, HeLa-EFNB1 cells displayed ∼100% more efficient attachment to fibronectin compared to HeLa-Ctrl cells (*p* < 0.0005; one-way ANOVA and Dunnett’s post-hoc test) (Fig. 2*A*). To test a functional requirement of EFNB1 proximity partners to EFNB1-dependent cell attachment to fibronectin, we selected 7 candidates based on molecular features. We included proteins potentially involved in pTyr-dependent signaling, such as Tyr kinases (EGFR, ROR2), as well as SH2 (PIK3R2, SHB) and PTB domain-containing proteins (EPS8). We also selected a protein potentially capable of engaging EFNB1’s PBM *via* its PDZ-domain (LIN7C) and a Ser/Thr kinase (TNIK), as Ser phosphorylation was reported to regulate ephrin-B-dependent functions (55). We tested whether their individual depletion using two different siRNAs led to changes of HeLa-EFNB1 cells’ ability to attach to fibronectin-coated plates. We found that depletion of TNIK or EGFR led to a consistent decrease in cell attachment of >50% compared to HeLa-EFNB1 cells treated with siCtrl (∗∗∗*p* < 0.0005, ∗∗∗∗*p* < 0.0001; one-way ANOVA and Dunnett’s post-hoc test) (Fig. 2*A*). We validated EGFR knockdown efficiency by Western blotting for each siRNA sequence (Fig. 2*B*), and decided to further pursue EGFR, as we and others recently reported its crosstalk with EPH receptor family members (33, 56, 57, 58).

To investigate the association between EFNB1 and EGFR, we performed GFP affinity purification from lysates of HEK293T cells overexpressing EFNB1-GFP (B1-GFP) or GFP alone (Fig. 2*C*). Remarkably, endogenous EGFR was detected in B1-GFP but not GFP complexes (Fig. 2*D*). To pinpoint which EFNB1 region is necessary for this association, we used GFP-tagged EFNB1 deletion mutants lacking the intracellular domain (dICD) or the PBM (dPBM) (Fig. 2*C*). We validated that both mutants are localized at the plasma membrane and are capable of activating EPHB3 (Supplemental Fig. S3). Importantly, we found that deletion of either EFNB1’s ICD or PBM significantly decreased EFNB1 association with EGFR (Fig. 2, *E* and *F*), indicating that EFNB1’s PBM of four amino acids is the minimal region required for its association with EGFR. Since PBMs are typically located at the C-terminus of proteins, we sought to eliminate a possible interference of the C-terminal GFP tag in the EFNB1-EGFR association. To achieve this, we used the recombinant intracellular domain of EFNB1 fused to a maltose-binding protein (MBP) tag at the N-terminus (MBP-B1-ICD) to pulldown EGFR from lysates of HEK293T cells overexpressing EGFR. We found that EGFR is pulled-down with MBP-B1-ICD but not with MBP-B1-ICD-dPBM (Supplemental Fig. S4). To test whether EFNB1’s PBM is required for EFNB1-dependent cell adhesion to fibronectin, we used a HeLa T-REx cell line stably overexpressing EFNB1-dPBM. We found that attachment of these cells to fibronectin is reduced by approximately 50% compared to EFNB1 WT expressing cells (*p* = 0.0004; one-way ANOVA and Dunnett’s post hoc test) and is comparable to the attachment of Hela-Ctrl cells (Fig. 2, *G* and *H*). Altogether, these results indicate that EFNB1’s PBM is required for the association with EGFR and EFNB1-dependent cell adhesion to fibronectin.

### EGFR Phosphorylates Tyrosine Residues Within EFNB1 PBM

**Fig. 3:**
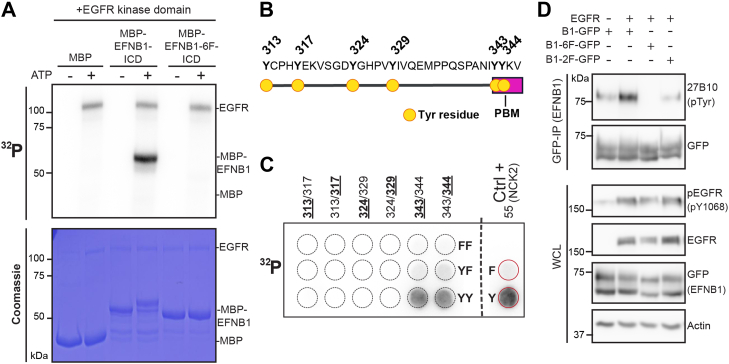
**EGFR phosphorylates tyrosine residues within EFNB1 PDZ-binding motif.***A*, *in vitro* radioactive kinase assay using recombinant intracellular domain (ICD) of EFNB1 (aa259–346) or EFNB1-6F, and recombinant kinase domain of EGFR. Incorporation of radioactive ^32^P was assessed by autoradiography. *B*, schematic representation of the last 34 aa of EFNB1 C-terminus. Position of the six Tyr residues is indicated on the top. *C*, *in vitro* kinase assay on peptide arrays displaying all Tyr residues in the intracellular domain of EFNB1. Positions of the Tyr residues tested in each peptide are indicated on the top. The row on the top displays negative control peptides, where all Tyr resides were replaced by Phe (FF). *Middle row* (YF) displays peptides with a single Tyr residue available (marked in *bold on top*). *Bottom row* contains peptides with both Tyr residues available (YY). *D*, Full-length EFNB1 fused to GFP (B1-GFP) or Y/F mutants were co-overexpressed with EGFR. Activation of EGFR was evaluated with a phospho-EGFR antibody (pEGFR) and EFNB1 Tyr phosphorylation was assessed using the pTyr antibody 27B10. B1-6F-GFP contains Phe replacing all six Tyr of EFNB1; B1-2F-GFP refers to Y343, 344F.

To explore a possible kinase–substrate relationship between EGFR and EFNB1, we performed a radioactive *in vitro* kinase assay using EGFR catalytic domain and recombinant EFNB1 intracellular domain (aa259–346) fused to MBP (MBP-B1-ICD). We detected a significant incorporation of [*γ-*^*32*^*P*] in EGFR and MBP-B1-ICD, while no signal was observed for MBP alone or MBP-B1-6F-ICD, a mutant where all six EFNB1 Tyr residues were replaced with Phe (Fig. 3*A*). These data confirmed the ability of EGFR to directly phosphorylate EFNB1. To further determine which of the EFNB1 Tyr residue(s) are directly phosphorylated by EGFR, we employed an array displaying 13aa peptides each encompassing one or two of the six Tyr residues of the EFNB1 intracellular domain (Fig. 3, *B* and *C* and Supplemental Table S2). We incubated the peptide array with EGFR catalytic domain and found that EFNB1 peptides displaying both Tyr343 and Tyr344, which are located within EFNB1 PBM, were phosphorylated (Fig. 3*C*). Interestingly, the signal from peptides displaying only one of Tyr343 or Tyr344 was much weaker, suggesting that EGFR preferentially phosphorylates this motif when both Tyr residues are available. Furthermore, we examined whether EGFR activation induces EFNB1 Tyr phosphorylation in cells. Co-transfection of EGFR led to a substantial Tyr phosphorylation increase in EFNB1 WT but not EFNB1 Y343, 344F (EFNB1-2F), or 6F (Fig. 3*D*). Taken together, these results suggest that EGFR directly phosphorylates EFNB1 Tyr343 and Tyr344 within the PBM.

### EFNB1 PBM Tyr Phosphorylation Disrupts its Association with EGFR and Blocks EFNB1-Dependent Cell Adhesion to Fibronectin

**Fig. 4:**
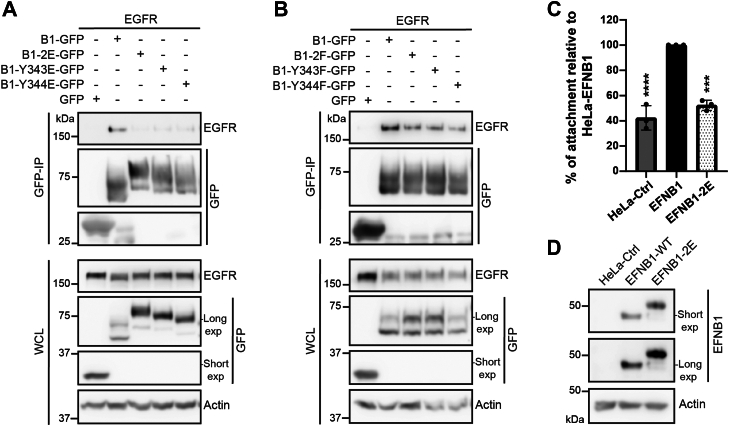
**EFNB1 PBM Tyr phosphorylation disrupts its association with EGFR and blocks EFNB1-dependent cell adhesion to fibronectin.***A* and *B*, Western blotting of EGFR following GFP-immunoprecipitation from cell lysates of HEK293T cells overexpressing EFNB1 fused to GFP (B1-GFP) or EFNB1 phosphomimetic (Y/E (Y343, 344E); *panel A*) or non-phosphorylable (Y/F (Y343, 344F); *panel B*) mutants, and EGFR. *C*, cell adhesion to fibronectin was assessed for HeLa T-REx cell lines that stably overexpress WT-EFNB1 or phosphomimetic mutant EFNB1-2E. Values were normalized relative to HeLa-EFNB1. Error bars indicate SD (∗∗∗*p* = 0.0001, ∗∗∗∗*p* < 0.0001; one-way ANOVA and Dunnett’s post-hoc test), n = 3. *D*, EFNB1 Western blot from HeLa T-REx lysates used in *panel C*.

To assess the consequence of EFNB1 PBM Tyr phosphorylation on its ability to associate with EGFR, we performed GFP affinity purifications from 293T lysates co-overexpressing EGFR and WT EFNB1-GFP or phosphomimetic mutants Y343E, Y344E, or Y343,344E (B1-2E-GFP). We found that EGFR is unable to associate with any of the EFNB1 phosphomimetic mutants, in contrast to its association with WT EFNB1 (Fig. 4*A*). Our data also revealed that the association with EGFR is decreased but still present with the EFNB1 non-phosphorylable (Y/F) mutants (Fig. 4*B*).

To test if EFNB1 PBM Tyr phosphorylation blocks EFNB1-dependent cell adhesion to fibronectin, we established a HeLa T-REx cell line stably expressing EFNB1-2E. We measured that these cells attached ∼50% less to fibronectin compared to WT EFNB1 (*p* = 0.0001; one-way ANOVA and Dunnett’s post-hoc test) (Fig. 4, *C* and *D*). Taken together, our data reveal that EGFR phosphorylates and associates with EFNB1 to regulate cell adhesion to fibronectin.

## Discussion

PPIs have been frequently studied using standard affinity-based purification approaches (59). However, studying PPIs in poorly soluble compartments such as the plasma membrane can be challenging (28). To overcome this, proximity labeling approaches have been developed and more recently resulted in the engineering of much more efficient mutant biotin ligases such as miniTurbo (miniT) (30). Here, we employed proximity biotinylation proteomics using miniT to identify EFNB1 proximity partners in a time-resolved fashion. This allowed us to identify a total of 104 proximity partners, from which 11 were classified as EPHB3-stimulation dependent, 49 as EPHB3-stimulation independent, and 44 as negatively modulated by EPHB3-stimulation. Consistent with other proximity labeling proteomics studies (28), ∼86% of our dataset represents putative partners that were not previously reported.

Previous studies have shown that Tyr phosphorylation in the ephrin-Bs intracellular domain leads to the assembly or disassembly of protein complexes (8, 9, 16, 17), a process known as “reverse” signaling (2). Ephrin-Bs were described as substrates for members of the SRC family of Tyr kinases, such as SRC and FYN (10, 11) and can also be phosphorylated following the activation of RTKs such as FGFR (60) and PDGFR (49). We found that EGFR directly phosphorylates EFNB1’s PBM, a sequence of four amino acids at its C-terminus (YYKV). Interestingly, we observed that EGFR preferentially phosphorylates this motif when both Tyr residues are available, as there was a significant reduction in phosphorylation when one of the two residues was replaced by Phe. This is in accordance with EGFR specificity for pTyr-primed substrates, which in most cases contain motifs with two adjacent Tyr residues (61). In cells, the recognition of pTyr-primed EFNB1 by EGFR may be a regulatory mechanism to integrate different pTyr-dependent signaling pathways downstream of EFNB1.

In addition, we found that EFNB1’s PBM phosphorylation on Tyr343 or Tyr344 disrupts its association with EGFR. There are many reports showing that Tyr, Ser, or Thr phosphorylation within the PBM of a given protein impairs its binding to targets (62). For example, Thr phosphorylation within the PBM of CACNG2 (stargazin) prevents the binding to PSD95 PDZ-domain (63). Another group observed a significant decrease in the binding affinity for the interaction between AFDN and its target JAG1 (jagged-1) when the PBM of the latter was Tyr phosphorylated (64). Also, in the context of EFNB1, it has been shown that Tyr phosphorylation within the PBM disrupts the interaction with the PDZ-domain of SDCBP (syntenin), a known EFNB1-binding partner (8). In addition, Songyang *et al*. identified a subset of PDZ-domains that preferentially select motifs containing aromatic residues. Remarkably, these PDZ domains preferred Phe but were also able to recognize Tyr residues (65). This provides an explanation for our observation of a sustained interaction between EGFR and EFNB1 Y343,344F mutants.

Ephrin-Bs are known to regulate in a cell-autonomous manner multiple types of cell adhesion events, including enhancement of integrin-mediated cell adhesion to extracellular matrix proteins, such as fibrinogen, laminin, and fibronectin (21, 22, 23). Importantly, Meyer and colleagues demonstrated that this enhancement in cell adhesion is abrogated when deleting the intracellular domain of ephrin-Bs, highlighting the importance of reverse signaling in this context (23). As expected, we observed an increase in cell adhesion when overexpressing WT EFNB1 in HeLa cells and found that knocking down EGFR, deleting EFNB1’s PBM or simulating Tyr phosphorylation within this motif abrogates this phenotype, indicating that the association between EGFR and EFNB1 is instrumental to recapitulate EFNB1-dependent cell adhesion to fibronectin. As dysregulation of cell-ECM interactions is a common feature in cancers (66), our study raises the possibility that EGFR and EFNB1 cooperate to regulate cell-matrix adhesion in cancer types where both proteins are overexpressed, possibly including brain, breast, and lung cancers (67, 68, 69, 70, 71, 72).

To facilitate the characterization of the EFNB1-EGFR association, we performed several GFP-mediated affinity purifications in settings where EFNB1 and EGFR were both overexpressed. In general, a drawback of studying PPIs in an overexpression setting is that the increased protein levels could lead to changes in protein interaction dynamics. Nevertheless, our detailed analysis of the EFNB1-EGFR association, combined with the previous observation of their association in an endogenous setting in primary human tonsil epithelial cells (33), significantly increases confidence in our observations.

For proximity labeling proteomics, we opted for a negative control displaying cytoplasmic compartmentalization (YFP-miniT-Flag). We previously observed that a plasma membrane-localized control was too stringent, leading to nonspecific labeling of biologically relevant proximity partners (34). However, we acknowledge that this cytoplasmic control could lead to a number of false negatives, for example, candidates that interact at the plasma membrane and then translocate to the cytoplasm and/or nucleus. This could be the case for STAT3, which can be activated and translocated to the nucleus following its association with phosphorylated EFNB1 at the plasma membrane (13). In this study, STAT3 was indeed identified with both EFNB1 and YFP-miniT fusions and did not pass our stringent 1% BFDR cutoff. Likely, other interesting proximity partners were also deemed as being false negatives. Nonetheless, the identification of 90 potential EFNB1 signaling effectors represents a valuable resource that can help the scientific community to better understand the molecular mechanisms underlying EFNB1-dependent processes and phenotypes.

## Data Availability

Mass spectrometry proteomics data have been deposited to the ProteomeXchange Consortium *via* the MassIVE partner repository (https://massive.ucsd.edu) with the dataset identifier MSV000096330. This article contains supplemental data.

## Supplemental Data

This article contains supplemental data.

## Conflict of Interest

The authors declare that they have no conflicts of interest with the contents of this article.
