# Supplementary material for: EGFR Phosphorylates and Associates with EFNB1 to Regulate Cell Adhesion to Fibronectin
Source: Mol Cell Proteomics. 2025 Jul 4;24(8):101027. doi: 10.1016/j.mcpro.2025.101027 (PMC12345308; doi:10.1016/j.mcpro.2025.101027)
Supplement: Supplementary data [file mmc6.pdf]

SUPPLEMENTARY DATA

EGFR phosphorylates and associates with EFNB1 to regulate cell adhesion to fibronectin.

Ana I. Osornio-Hernández[1,2,3], François J. M. Chartier[2,3], Tim L. Schuehle[2,3], Sara L. Banerjee[2,3,4], Sabine Elowe[2,3,5], Patrick Laprise[1,2,3], Andrew Freywald[6], Mélanie Laurin[1,2,7] and Nicolas Bisson[1,2,3,7]

[1]Department of Molecular Biology, Medical Biochemistry and Pathology, Faculté de Médecine, Université Laval, Québec, QC, Canada

[2]Centre de recherche du Centre Hospitalier Universitaire (CHU) de Québec-Université Laval, Division Oncologie, Québec, QC, Canada

[3]PROTEO and Centre de recherche sur le cancer de l'Université Laval, Québec, QC, Canada

[4]Present address: Neural Circuit Development Research Unit, Institut de Recherches Cliniques de Montréal (IRCM), and Division of Experimental Medicine, McGill University, Montréal, QC, Canada

[5]Department of Pediatrics, Université Laval, Québec, QC, Canada

[6]Department of Pathology and Laboratory Medicine, College of Medicine, University of Saskatchewan, Royal University Hospital, Saskatoon, SK, Canada

[7]Corresponding authors: melanie.laurin@crchudequebec.ulaval.ca; nick.bisson@crchudequebec.ulaval.ca

Running title: EGFR and EFNB1 regulate cell adhesion.

Contents

Figure S1: Dot plot displaying EFNB1 proximity partners.

Figure S2: GO enrichment analysis.

Figure S3: Validation of EFNB1 deletion mutants.

Figure S4: The intracellular domain of EFNB1 pulls down EGFR.

Table S1: siRNA target sequences (Excel file).

Table S2: Peptide sequences and positions for peptide arrays (Excel file).

Table S3: SAINT results for EFNB1 proximity labeling in steady state (Excel file).

Table S4: SAINT results for EFNB1 proximity labeling under EPHB3-stimulation (Excel file).

Table S5: Protein and peptide identification data (Excel file).

Data availability: Mass spectrometry proteomics data have been deposited to the ProteomeXchange Consortium via the MassIVE partner repository (https://massive.ucsd.edu) with the dataset identifier MSV000096330.

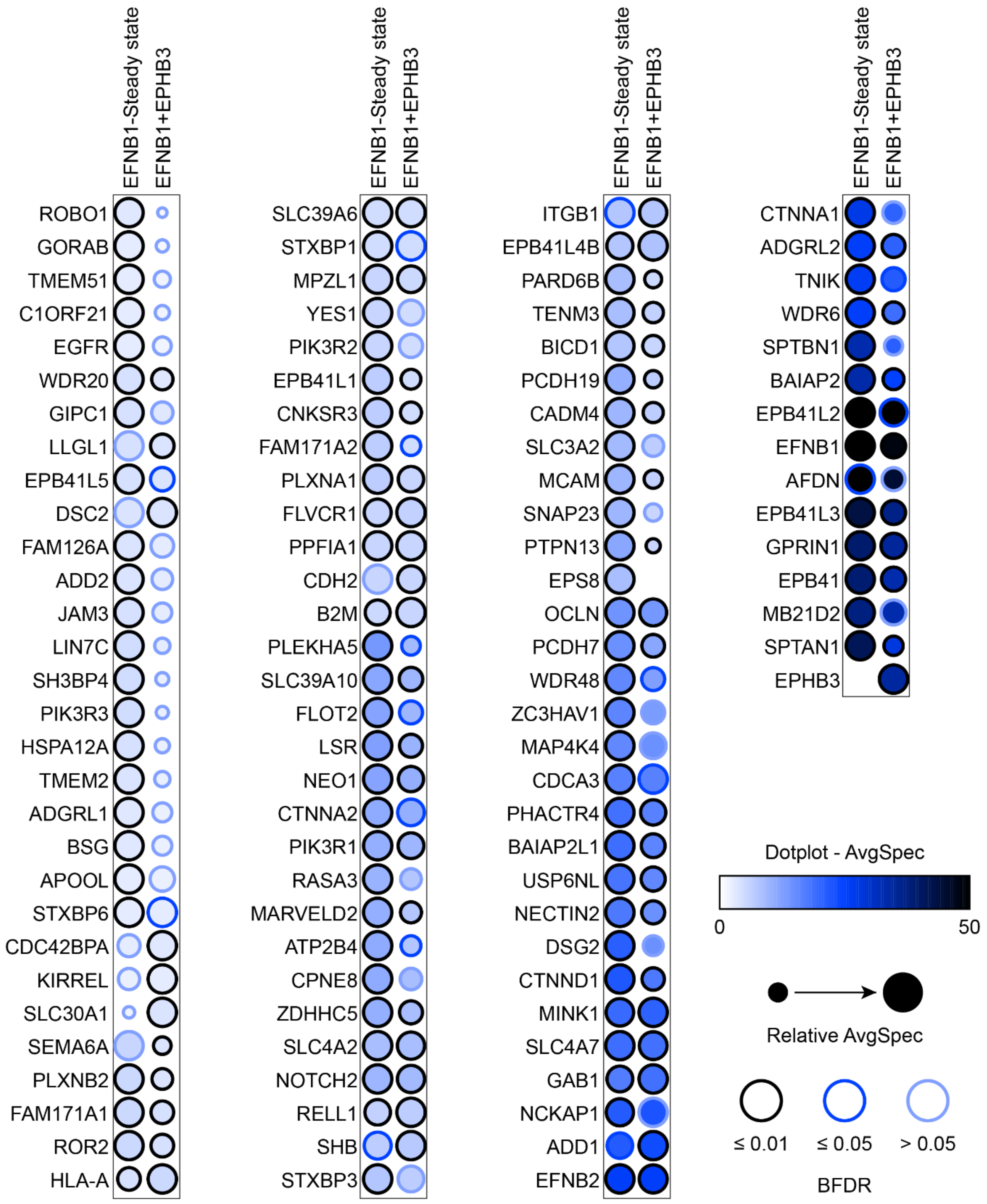

Supplementary figure S1: Dot plot displaying EFNB1 proximity partners.

Dot plot representation of changes in abundance between steady state and EPHB3-stimulation for all identified EFNB1 proximity partners.

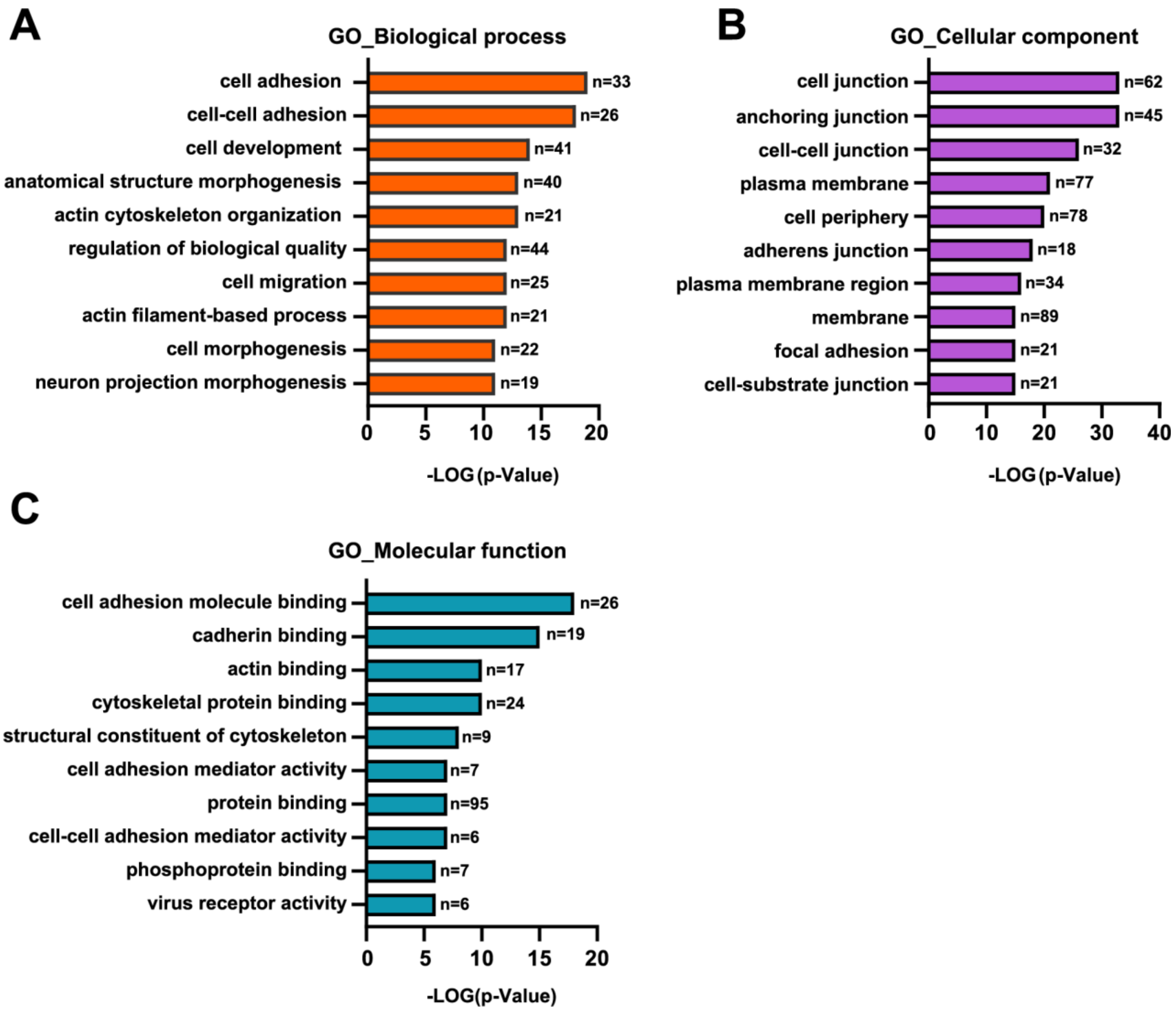

Supplementary figure S2: GO enrichment analysis.

Enrichment analysis of EFNB1 proximity partners showing the top 10 most significantly enriched terms for **(A)** Biological process, **(B)** Cellular component and **(C)** Molecular function.

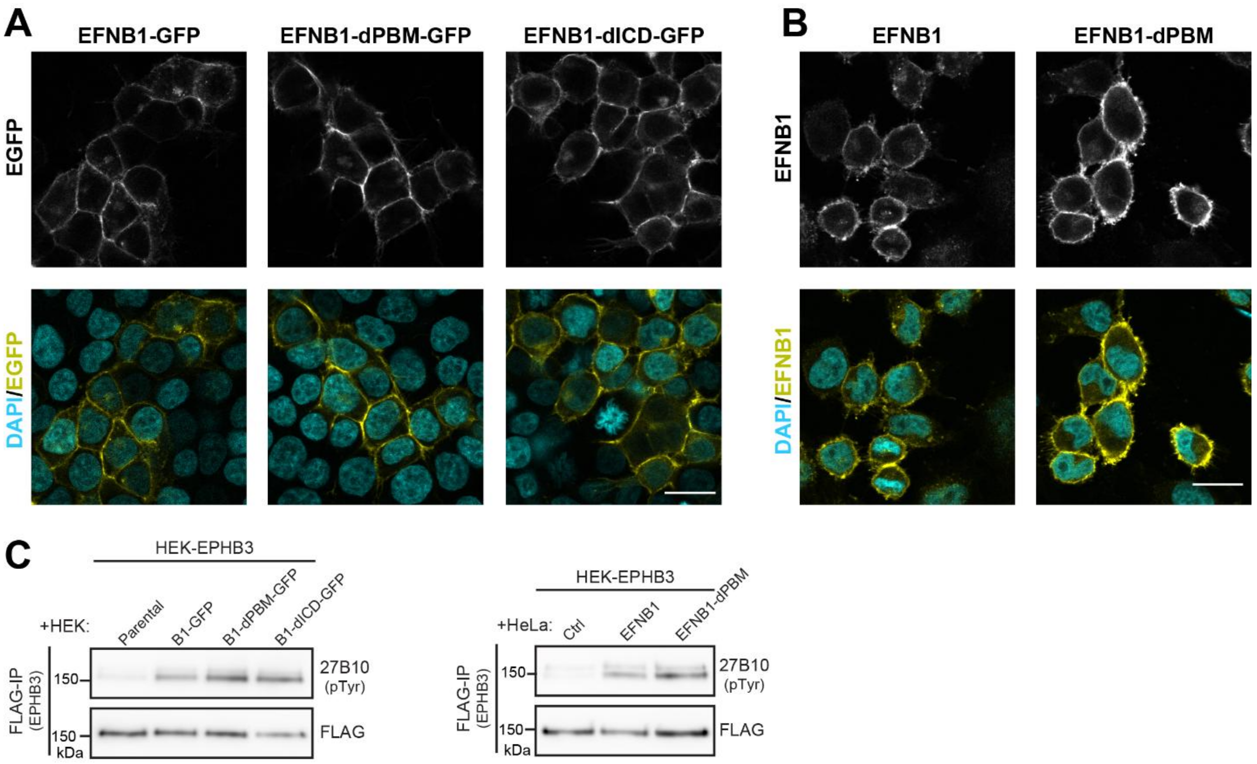

Supplementary figure S3: Validation of EFNB1 deletion mutants.

(A) Subcellular localization of EFNB1 GFP-tagged fusions in HEK293 cells was assessed by confocal microscopy using the signal from GFP. Scale bar represents 20 µm. **(B)** Subcellular localization of EFNB1 and EFNB1-dPBM in HeLa T-REx cells was evaluated by immunofluorescence using anti-EFNB1 antibodies. Scale bar represents 20 µm. **(C)** HEK293 T-REx cells overexpressing EPHB3-Flag were mixed for 20 min with cells overexpressing EFNB1 GFP-tagged fusions (left) or EFNB1 untagged constructs (right). EPHB3 was then immunoprecipitated (FLAG-IP) and Tyr phosphorylation was assessed using the anti-pTyr antibody 27B10.

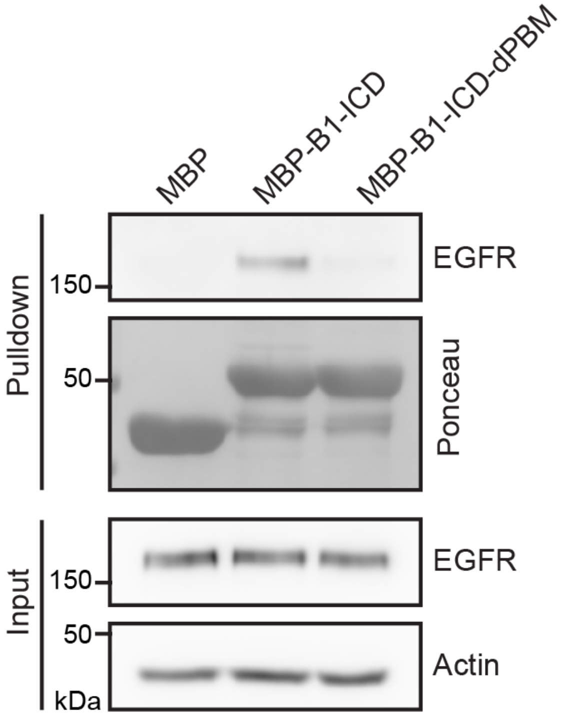

Supplementary figure S4: The intracellular domain of EFNB1 pulls down EGFR.

The intracellular domain of EFNB1 (aa259-346) fused to MBP in the N-terminus (MBP-B1-ICD) or PBM deletion mutant (MBP-B1-ICD-dPBM) were used to pulldown EGFR from lysates of HEK293T cells overexpressing EGFR.
